# The Rice Peptide Transporter *OsNPF7.3* Is Induced by Organic Nitrogen, and Contributes to Nitrogen Allocation and Grain Yield

**DOI:** 10.3389/fpls.2017.01338

**Published:** 2017-08-02

**Authors:** Zhongming Fang, Genxiang Bai, Weiting Huang, Zhixin Wang, Xuelu Wang, Mingyong Zhang

**Affiliations:** ^1^Center of Applied Biotechnology, Wuhan Institute of Bioengineering Wuhan, China; ^2^National Key Laboratory of Crop Genetic Improvement, Huazhong Agricultural University Wuhan, China; ^3^Key Laboratory of South China Agricultural Plant Molecular Analysis and Genetic Improvement and Guangdong Provincial Key Laboratory of Applied Botany, South China Botanical Garden, Chinese Academy of Sciences Guangzhou, China

**Keywords:** nitrogen, transporter, rice, tiller number, grain number

## Abstract

Nitrogen use efficiency is important for the development of sustainable agriculture. Plants have different transporters to facilitate nitrogen uptake and internal distribution. This study demonstrates that the peptide transporter *OsNPF7.3* enhances nitrogen allocation and increases grain yield in rice. OsNPF7.3 is a member of the nitrate transporter 1/peptide transporter family (NPF) and is localized in the vacuolar membrane. Its expression is higher in the lateral roots and stems. Its transcripts concentrate in the vascular bundle and significantly regulated by organic nitrogen sources. The RNAi lines of *OsNPF7.3* affect plant growth and cause amino acids to accumulate in leaf sheaths and decrease in the leaf blades. At later stages of reproductive growth, nitrogen degradation accelerates in the leaves of plants over-expressing *OsNPF7.3* and the nitrogen is translocated to grains. The tiller numbers, panicles per plant, filled grain numbers per panicle, and grain nitrogen content of the *OsNPF7.3* over-expressing plant were more than that of wide type. The elevated gene expression in *OsNPF7.3* could enhance nitrogen utilization efficiency in rice paddy.

## Introduction

Nitrogen use efficiency is important for the development of sustainable agriculture (Xu et al., 2012). Plants have different transporters to facilitate nitrogen uptake and internal distribution (Rentsch et al., 2007). NPF family (previous called as PTR/NRT1 family) could uptake and translocation of nitrate or small peptides in higher plants. *Arabidopsis* and rice have at least 53 and 93 NPF genes, respectively (Tsay et al., 2007; Zhao et al., 2010; Léran et al., 2014).

Only a few members of this family have been characterized for transport function. For *Arabidopsis*, AtPTR1, AtPTR2, AtPTR3, and AtPTR5 could transport di- and tripeptide (Tegeder and Rentsch, 2010). AtNRT1.1 to 1.9 could transport nitrate with low affinity (Dechorgnat et al., 2011). Some transport other substrates like carboxylates, auxin, or abscisic acid (ABA) (Tsay et al., 2007; Krouk et al., 2010; Kanno et al., 2012). AtPTR1 participates in root nitrogen uptake (Komarova et al., 2008), while AtPTR2 is involved in flower and seed development (Song et al., 1997). *AtPTR5* and *AtPTR6* are associated with pollen expression, and AtPTR2 and AtPTR6 are active during senescence (Komarova et al., 2008; Weichert et al., 2012). AtNRT1.1–1.9 participate in several key steps of nitrate uptake or allocation (Xu et al., 2012).

Among the rice NPF family, only nine have been studied. OsNRT1 (OsNPF8.9; Lin et al., 2000) is a low affinity nitrate transporter. *SP1* (*OsNPF4.1*; Li et al., 2009) determines panicle size, but its substrate is still unknown. It was found that the over-expression of *OsPTR9* (*OsNPF8.20*) enhances ammonium uptake and increases grain yield in rice (Fang et al., 2013). OsNPF2.4 could transport nitrate with the low-affinity acquisition and transportation (Xia et al., 2015). The *OsNRT1.1B* (*OsNPF6.5*) *Indica* variant enhances nitrate uptake, and could increase nitrogen use efficiency (Hu et al., 2015). Rice nitrate transporter *OsNPF2.2* is beneficial to root-to-shoot nitrate transport and relates to vascular development (Li et al., 2015). Recently, the mutant of the low-affinity nitrate transporter *OsNPF7.2* retards rice growth under high nitrate supply (Hu et al., 2016), and *OsPTR7* (*OsNPF8.1*) is involved in dimethylarsenate accumulation in rice grain (Tang et al., 2017).

*OsPTR6* (*OsNPF7.3*) transports the di/tripeptides Gly-His and Gly-His-Gly (Ouyang et al., 2010). The over-expression of *OsNPF7.3* in rice could enhance plant growth (Fan et al., 2014). Nevertheless, the regulation of its expression and its biological function in rice is very limited. Plant membrane transporters can be used to enhance crop yields (Schroeder et al., 2013). Therefore, the systematic study of the function of *OsNPF7.3* will elucidate its effects on the agronomic traits of rice. This present study indicates that *OsNPF7.3* is an organic nitrogen-regulated vacuolar membrane peptide transporter and was showed to contribute to nitrogen allocation and grain yield in rice.

## Materials and Methods

### Acquisition of the Transgenic Rice of *OsNPF7.3*

To analyze the *OsNPF7.3* promoter, a 2136 bp fragment upstream of *OsNPF7.3* open reading frame (ORF) was amplified by PCR using the primers shown in Supplementary Table 1, and inserted in front of the β-glucuronidase (GUS) coding region in pCAMBIA1301^1^ with *EcoR* I and *Nco* I to make the *pOsNPF7.3*-*GUS* plasmid. To construct *OsNPF7.3-*overexpressing plants, a 1782-bp *OsNPF7.3* ORF was inserted downstream of the *35S* promoter in pCAM1301 with *Bgl* II and *Afl* II, producing the *p35S*-*OsNPF7.3* plasmid. To generate the *OsNPF7.3*-RNAi construct, two 314-bp fragments of the *OsNPF7.3* cDNA were amplified using the primers listed in Supplementary Table 1 and transferred downstream of the *Ubi-1* promoter in vector pTCK303 (Wang et al., 2004) with *BamH*I/*Kpn*I and *Spe*I/*Sac*I, respectively, to generate the *OsNPF7.3*-RNAi vector. To produce *OsNPF7.3*-GFP, the *OsNPF7.3* ORF was amplified by PCR using the primers listed in Supplementary Table 1. The 1779-bp amplified *OsNPF7.3* ORF (lacking the stop codon) was cloned in front of the green fluorescent protein (GFP) coding region in the pCAM1302 vector with *Bgl* II and *Spe* I and the final *pOsNPF7.3-GFP* construct was created. All of the plasmid constructs were transferred into *Agrobacterium* strain *EHA*105, and then transformed into *japonica* rice variety Zhonghua 11 (ZH11) by *Agrobacterium*-mediated transformation method (Hiei et al., 1997). T2 or T3 transgenic lines were selected using real time-PCR. The corresponding primers are indicated in Supplementary Table 1.

### Subcellular Localization of OsNPF7.3

To understand the subcellular localization of OsNPF7.3, p35S-*OsNPF7.3*-*GFP*, and p35S-*GFP* were introduced into rice protoplasts. These were prepared and transformed according to previously protocols (Zhang et al., 2011). Protoplasts were isolated from leaf sheaths of rice seedlings after sowing for 7–15 days. *OsNPF7.3*-GFP under control of the 35S promoter was introduced into tobacco epidermal cells using transferred into tobacco by *Agrobacterium*-mediated transformation. The green fluorescence of GFP and coexpressed membrane marker AtTPK-mkate (Voelker et al., 2006) were observed using a confocal laser scanning microscope (Leica SP8 AOBS, Wetzlar, Germany).

### Rice Culture Conditions

Hydroponic experiments were conducted with filled sand under glasshouse using the basic rice culture solution (Yoshida et al., 1976). To analyze *OsNPF7.3* expression in the presence of different N sources, promoter-GUS transgenic seedlings and ZH11 were grown in the basic nutrient solution for 3 weeks using 1 mM NH_4_NO_3_ as the N source. They were then transferred to basic nutrient solution without N where they remained for 3 days. The N-starved seedlings were transferred to basic nutrient solutions supplemented with one of the following as the sole N source: 0.5 mM NaNO_3_; 2.0 mM NaNO_3_; 5.0 mM NaNO_3_; 0.25 mM (NH_4_)_2_SO_4_; 1.0 mM (NH_4_)_2_SO_4_; 2.5 mM (NH_4_)_2_SO_4_; 0.25 mM NH_4_NO_3_; 1.0 mM NH_4_NO_3_; 2.5 mM NH_4_NO_3_; 2% peptone; 2 mM Gln; or 2 mM Glu. Samples were harvested at different times for histochemical GUS staining and RNA extraction. *OsNPF7.3* transgenic plants on sand were grown in basic nutrient solution supplemented with 1.0 mM NH_4_NO_3_. The nutrient solution was renewed every 3 days. The greenhouse condition is 32°C for sodium lamp 400w 14 h in the daytime and 25°C for dark 10 h in the evening. The paddy rice grew at season June to October at rice experimental base of Huazhong Agricultural University under fertilizer (N/P/K = 19%/7%/14%) 180 kg/hm^2^, and the nitrogen level of soil in rice paddy is 90 mmol/kg. Generally, the number of rice plants was 30 for each experiment and the planting density was 19.98 × 19.98 cm. For the field yield trials, the number of rice plants was 100 for each OE line and ZH11.

### GUS Staining

Histochemical GUS staining was performed according to Jefferson (1987). The stained materials were observed using light or fluorescence microscopy. To make sections, the stained tissues of root, stem, leaf, and panicle were rinsed and fixed in FAA (formalin-acetic acid-70% ethanol [1:1:18]) at 4°C for 24 h, gradually dehydrated with ethanol for 15 min each time, and washed two times with 100% ethanol for 30 min each time. Finally, embedded in Spurr resin, and then sectioned. The sections were observed using Zeiss Axio Imager M2 (Carl Zeiss AG, Oberkochen, Germany).

### Nitrogen, Amino Acids, and Chlorophyll Contents; Photosynthesis Analysis

Total free amino acid concentration was measured by the ninhydrin method (Fang et al., 2013). Single free amino acid concentration was measured by HPLC method using amino acid analyzer L-8800 HITACHI. The samples were prepared as follows: 1 g rice tissue was mixed, then was soaked in 80% ethanol 10 ml at 80°C water bath for 20 min, and the water bath was repeated two times. The collected extracts were placed at 80°C dry bath to remove ethanol, and 1 ml 0.5 M NaOH was used to dissolve the substance, then centrifuged with 14000 rpm for 15 min. The supernatant was filtered with filter membrane 2 μm, and the filtrates 0.8 ml was tested with the amino acid analyzer. Total nitrogen content and total protein content were determined using the semi-micro Kjeldahl method by using a nitrogen analyzer (Smart Chem 200, Westco, Italy). Total soluble protein were pyrolyzed then measured by the Coomassie brilliant blue G250 method (Sedmak and Grossberg, 1977). Chlorophyll content was measured using the technique of Dhindsa et al. (1981). Photosynthesis was measured by portable photosynthetic apparatus LI-6400 (LI-COR Company, America). Nitrogen utilization efficiency was determined from the formula [grain yield (g)/grain nitrogen content (g) + straw nitrogen content (g)] × 100%.

### Quantitative Real-Time PCR

Total RNA was extracted using TRIzol reagent (Invitrogen, Beijing, China). First-strand cDNA were synthesized using oligo (dT) primers and MLV reverse transcriptase (TaKaRa Bio, Beijing, China). Quantitative real-time PCR was performed using SYBR Green mix (TaKaRa Bio, Beijing, China) and monitored with the 7500 RT qPCR system (Applied Biosystems, Foster City, CA, United States). The primers used for qPCR are listed in Supplementary Table 1.

### Statistical Analysis

For all treatments, Duncan’s test was run using one-way ANOVA. Significance was accepted at *P* < 0.05 and *P* < 0.01.

## Results

### OsNPF7.3 Is a Vacuolar Membrane-Localized Member of the NPF Family

**FIGURE 1:**
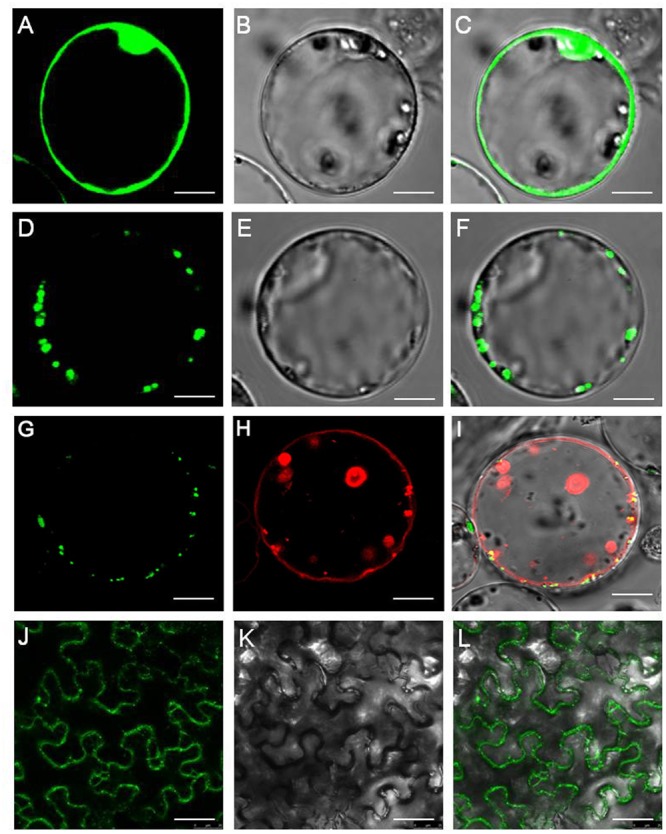
OsNPF7.3 is located in the rice vacuolar membrane. **(A–C)** Free GFP expression in rice protoplasts. **(D–F)** *OsNPF7.3-GFP* expression in rice protoplasts. **(G–I)** Fluorescence of OsNPF7.3-GFP coexpressed with the vacuolar membrane marker AtTPK-mkate in transiently transformed rice protoplasts. **(J–L)** Fluorescence of OsNPF7.3-GFP expressed in tobacco epidermal cells. Presented here are fluorescence images of GFP **(A,D,G,J)**, corresponding bright-field images **(B,E,K)**, AtTPK-mkate fluorescence images **(H)** and merged images **(C,F,I,L)**. Bars = 5 μm for **(A–L)**.

First, we attempted to determine its intracellular localization of peptide transporter OsNPF7.3. The transient expression of *35S*: *OsNPF7.3*-GFP in rice protoplast produced green fluorescence at the vacuolar membrane (**Figures 1D–F**), but for the *35S*-GFP control it occurred at the plasma membrane and nucleus (**Figures 1A–C**). To further determine the subcellular localization of OsNPF7.3, OsNPF7.3-GFP fusion protein (under the control of the CMV *35S* promoter) was transiently coexpressed with the vacuolar membrane marker AtTPK-mkate in rice protoplasts. The results showed that the OsNPF7.3-GFP fusion protein signal was localized at the vacuolar membrane and overlap with the vacuolar membrane marker signal (**Figures 1G–I**). In addition, it showed OsNPF7.3-GFP localized to the membrane system using *Agrobacterium* carried OsNPF7.3-GFP injection to tobacco leaf (**Figures 1J–L**). It was also observed that OsNPF7.3 has 12 transmembranes structure (TM). Overall, these data indicate that OsNPF7.3 is vacuolar membrane-localized peptide transporter.

### The Expression of *OsNPF7.3* Is Higher in Lateral Roots and Stems, and Is Significantly Regulated by Organic Nitrogen

**FIGURE 2:**
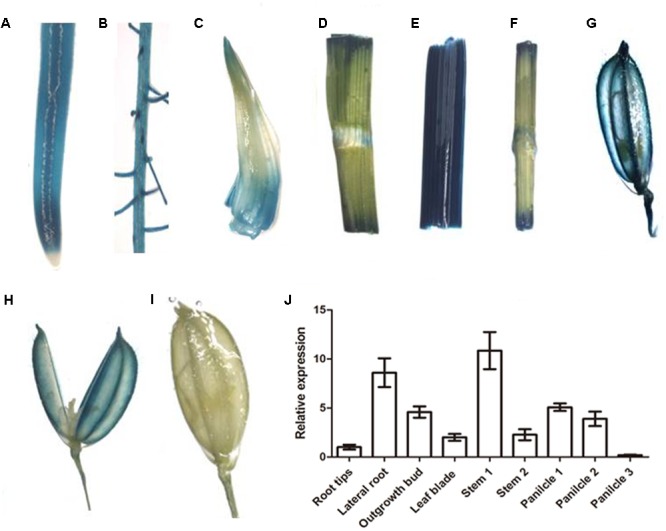
The expression analysis of *OsNPF7.3* in rice promoter-GUS and WT plants. GUS expression under the control of the *OsNPF7.3* promoter was detected in root tip **(A)**, lateral roots **(B)**, outgrowth bud **(C)**, leaf blade **(D)**, stem connection root to leaf **(E)**, stem connection leaf to panicle **(F)**, young panicle **(G)**, opened young panicle **(H)**, and filled panicle **(I)**. And the expression analysis of *OsNPF7.3* was detected by real time-PCR **(J)**.

*OsNPF7.3* is expressed in most of organs, as shown in transgenic rice plants with the promoter of *OsNPF7.3* driving *GUS* expression (**Figure 2**). In roots, GUS staining was observed in root tip (**Figure 2A**), and lateral roots (**Figure 2B**). In the seedling shoots, GUS staining is noted in the outgrowth buds (**Figure 2C**), leaf blade (**Figure 2D**), stems (**Figures 2E,F**). Furthermore, we found that *OsNPF7.3* is expressed in young panicles (**Figures 2G,H**), but it is not expressed in anther of panicle (**Figure 2H**) and filled grains (**Figure 2I**). In addition, the expression of *OsNPF7.3* is higher in lateral root and stem by real time-PCR in further experiment (**Figure 2J**).

**FIGURE 3:**
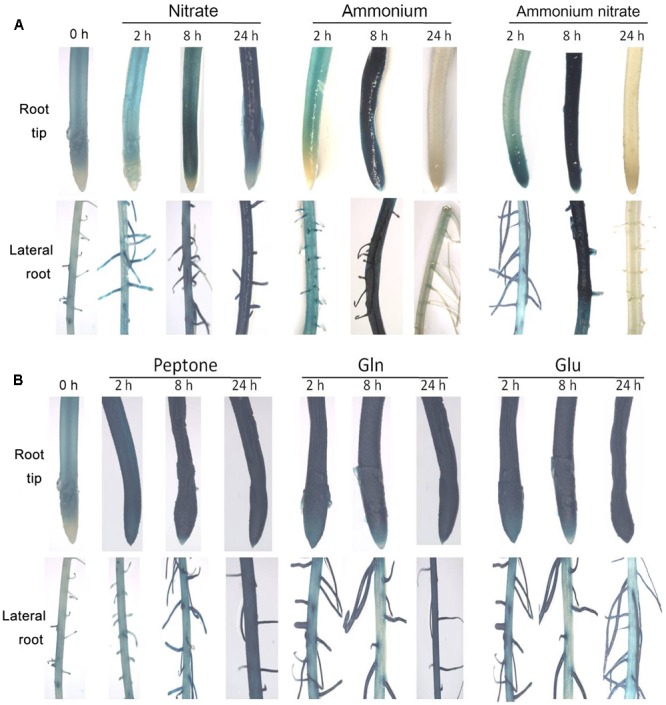
The expression of *OsNPF7.3* is regulated by both inorganic and organic nitrogen. Promoter-GUS transgenic seedlings were grown under glasshouse for 3 weeks in basic nutrient solution with 1 mM NH_4_NO_3_ as the N source then transferred to N-free basic nutrient solution for 3 days (N starvation). The N-starved seedlings were transferred to basic nutrient solution supplemented with 2.0 mM NaNO_3_ or 1.0 mM (NH_4_)_2_SO_4_ or 1.0 mM NH_4_NO_3_ **(A)** or supplemented with 2% peptone or 2.0 mM Gln or 2.0 mM Glu **(B)** as the N sources under glasshouse. The greenhouse condition is 32°C for sodium lamp 400w in the daytime and 25°C for dark in the evening.

We produced promoter-GUS transgenic rice plants to determine the transcriptional response of *OsNPF7.3* to various nitrogen treatments. Promoter-GUS plants were transferred to various nitrogen source solutions to monitor if the GUS staining is induced. GUS staining was noted deeply in the root tip and lateral roots at 24 h with the nitrate treatment, but at 8 h with the ammonium and ammonium nitrate treatments (**Figure 3A**). Similarly, the expression of *OsNPF7.3* was significantly increased with the nitrate between 0 and 24 h, but decreased in ammonium and ammonium nitrate treatments at 24 h by real time-PCR (Supplementary Figures 1A,B). To systematically characterize the regulation of *OsNPF7.3* expression by other nitrogen sources, GUS staining was performed using the organic nitrogen treatments. Intense GUS staining (**Figure 3B**) and increased expression level of *OsNPF7.3* (Supplementary Figures 1C,D) at 2, 8, and 24 h was observed where organic nitrogen (peptone, Gln, or Glu) was the sole nitrogen source.

**FIGURE 4:**
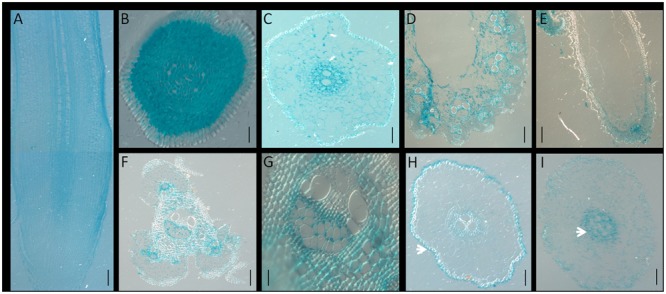
The expression of *OsNPF7.3* is enhanced in the parenchyma and vascular bundle cells. Longitudinal **(A)** and transverse **(B–I)** section analysis of p*OsNPF7.3*-*GUS* plant with GUS staining. GUS staining revealed that *OsNPF7.3* is expressed in root parenchyma cells **(A)**, root procambia **(B)**, and root vascular bundles **(C)**, leaf vascular bundles **(D)**, glume epidermis **(E)**, and stem vascular bundles **(F,G)**. Ammonium nitrate treatment **(H)**, and peptone treatment **(I)**. Bars = 20 μm **(B,G)**, 50 μm **(C–F,H,I)**, 500 μm **(A)**.

To determine the tissue-specific expression pattern of *OsNPF7.3*, cross- or longitudinal sections of the GUS-stained organs were made (**Figure 4**). Sectioning confirmed that higher expression of *OsNPF7.3* was observed in the cortex parenchyma and the vascular bundle of the root (**Figures 4A–C**), the vascular bundle of the leaf (**Figure 4D**), the glume epidermis (**Figure 4E**), and the vascular bundle of the stem (**Figures 4F,G**). It was revealed that *OsNPF7.3* expressed in the cortex parenchyma cells in the root by transverse sectioning (**Figures 4B,C**), and the parenchyma and sieve cells of the vascular bundle in the elongation zone (**Figure 4C**). The expression level of *OsNPF7.3* was also observed in the parenchyma and sieve cells of the vascular bundles in the leaf and stem but only in the epidermal cells of the glume (**Figures 4D–G**). When the roots were treated with peptone, sectioning also showed that GUS staining obviously intensified in the epidermal vascular bundle (**Figure 4I**) from epidermis (**Figure 4H**). Overall, these observations show that *OsNPF7.3* is expressed higher in the parenchyma cells adjacent to the veins and sieve cells in response to organic nitrogen application, and this may suggest that *OsNPF7.3* contributes to nitrogen allocation.

### Over-Expression of *OsNPF7.3* Enhance Biomass by Increasing Amino Acids and Total Nitrogen

To determine the function of *OsNPF7.3* in rice plants, we generated *OsNPF7.3*-RNAi transgenic rice plants (RNAi) under the control of the rice *Ubi-1* promoter. Sixty-five independent *OsNPF7.3*-RNAi T0 lines were obtained, and we chose five homozygous T2 lines for transcript level measurement, the result showed that the *OsNPF7.3* transcript levels of Ri-D1 to Ri-D5 are clearly reduced in leaves (Supplementary Figure 2), but *OsNPF7.3* expression is not decreased in the leaves of the null segrigant, which are separated from *OsNPF7.3*-RNAi (Ri-S1 to Ri-S5) lines (Supplementary Figure 2). We then selected the lines Ri-D1, Ri-D2, Ri-D3 for subsequent tests and labeled them Ri1, Ri2, and Ri3, respectively. To study the effects of increased *OsNPF7.3* expression, *OsNPF7.3* over-expressing (OE) rice was constructed under the control of the 35S promoter. Forty-eight independent OE T0 lines were obtained, and we chose five homozygous T2 lines for transcript level determination. The result showed that the transcript levels is quietly high in leaves of OE-D1, OE-D3, and OE-D5 (Supplementary Figure 2), and they were used for subsequent tests and labeled them OE1, OE2, and OE3, respectively.

**FIGURE 5:**
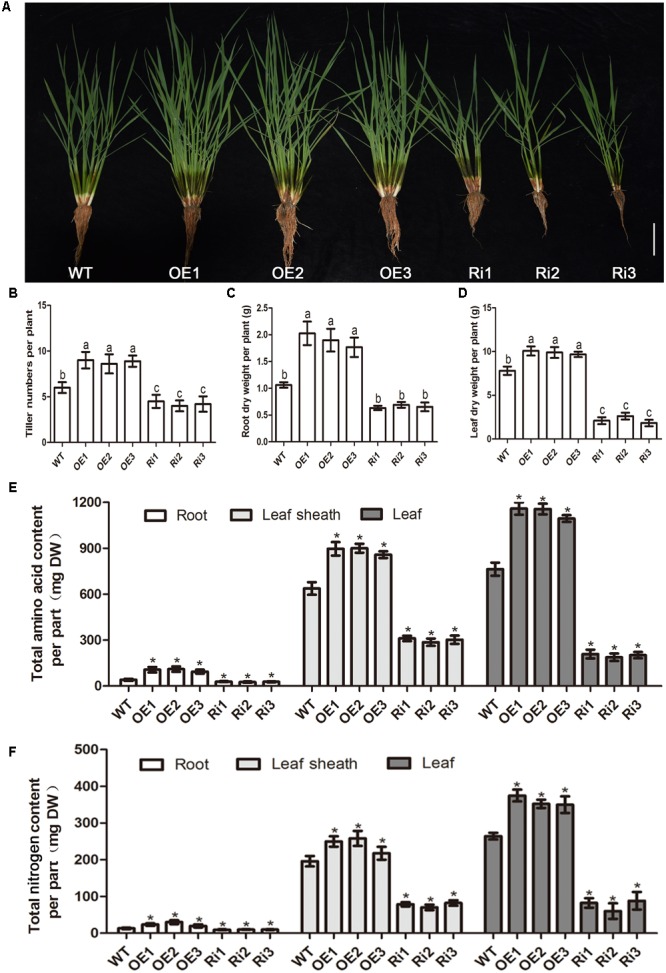
*OsNPF7.3* affects biomass and nitrogen allocation from root to leaf. Phenotype **(A)**, tiller number **(B)**, root dry weight per plant **(C)**, leaf dry weight per plant **(D)**, free amino acid content per organ **(E)**, and total nitrogen content per organ **(F)** were measured from 2 months sand cultured seedlings using basic nutrient solution supplemented with 1.0 mM NH_4_NO_3_ as the N source under glasshouse. The greenhouse condition is 32°C for sodium lamp 400w 14 h in the daytime and 25°C for dark 10 h in the evening. The OE1-OE3 depicts *OsNPF7.3*-overexpressing lines, Ri1-Ri3 depicts *OsNPF7.3*-RNAi lines, and WT depicts wild-type. Error bars depict the SD from three independent experiments using mixed samples from three lines. Different letters in the columns indicate a significant difference of *P* < 0.05 according to Duncan’s test. ^∗^Statistically significant differences were calculated by the Duncan’s multiple range test at the 5% level, when compared with wild-type rice (WT).

Since nitrogen regulates *OsNPF7.3* expression, we tested the responses of *OsNPF7.3* transgenic plants raised on sand with 1.0 mM NH_4_NO_3_. The results indicated that the tiller numbers of OE lines were higher compared with that WT, but lower of these RNAi lines (**Figures 5A,B**). The root dry weight per plant and leaf dry weight per plant of three OE seedlings were apparently higher than those of WT (**Figures 5C,D**). Inversely, down-expression of *OsNPF7.3* decreased those characteristics (**Figures 5A–D**). To investigate why *OsNPF7.3* affects rice growth, we measured the free amino acid content (**Figure 5E**) and total nitrogen content in the roots, leaf sheaths and the leaves of RNAi and OE lines. It showed that the free amino acid content per organ and total nitrogen content per organ of RNAi plants were lower than those of WT (**Figure 5F**). Nevertheless, free amino acid content and total nitrogen content of OE plants were relatively higher in all organs (**Figures 5E,F**). Furthermore, we found that sole free amino acid concentration (Asp, Gly, Ala, Cys, Val, Leu, Tyr, Phe, His, and Arg) was higher in the leaf sheath of RNAi lines, resulting in accumulation of total free amino acid concentration in leaf sheath (Supplementary Table 1). However, total free amino acid concentration was not changed in root and leaf of the RNAi lines, but increased in root and leaf of OE lines (Supplementary Table 1). Therefore, *OsNPF7.3* could affect nitrogen allocation from the roots to the leaves for rice growth.

### *OsNPF7.3* Recycles N from the Leaf to the Panicle

**FIGURE 6:**
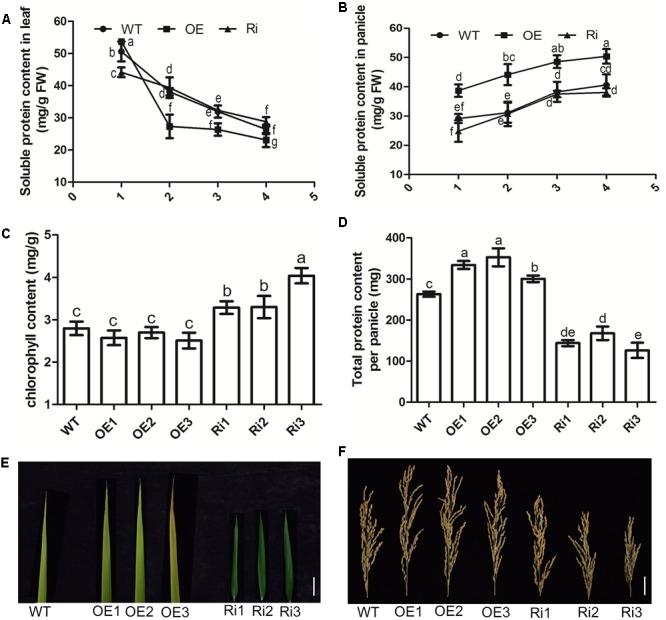
*OsNPF7.3* affects leaf senescence and nitrogen remobilization from leaf to panicle. Soluble protein contents in leaf **(A)**, and in panicle **(B)** at plant booting stage (1, 90 days after sowing), heading stage (2, 100 days after sowing), filling stage (3, 110 days after sowing) and mature stage (4, 120 days after sowing). Chlorophyll content at the mature stage **(C)**, total protein content per panicle **(D)**, the flag leaves at the mature stage **(E)**, and the panicles at the mature stage **(F)** were measured in paddy-grown plants. The paddy rice grew at season June to October at rice experimental base of Huazhong Agricultural University under fertilizer (N/P/K = 19%/7%/14%) 180 kg/hm^2^. Error bars depict the SD from three independent experiments using three mixed lines **(A,B)** or three separate lines **(C,D)**. Different letters in the columns indicate a significant difference of *P* < 0.05 according to Duncan’s test.

To investigate the role of *OsNPF7.3* in N transport and recycling during leaf senescence and grain filling, some indices of N recycling were measured. From the data, we found that the soluble protein content gradually decreased in the leaves of the OE lines during the reproductive stage (**Figure 6A**). This factor declined faster in the *OsNPF7.3* OE lines than WT and RNAi lines (**Figure 6A**). More importantly, the soluble protein content in the panicles was obviously higher in *OsNPF7.3* OE lines than the WT at all reproductive stages (**Figure 6B**). Chloroplast degradation accompanied the decrease in leaf protein content, so we measured the chlorophyll content of different transgenic plants. The results showed that the chlorophyll content accumulated in the RNAi lines (**Figure 6C**), and that the total grain protein per panicle increased in the *OsNPF7.3* OE lines but decreased in the RNAi lines (**Figure 6D**). Based on leaf phenotype observations, leaf senescence in the *OsNPF7.3* OE lines was accelerated but slowed down in the RNAi lines in the later reproductive stages (**Figure 6E**). Grain numbers increased in the *OsNPF7.3* OE lines but decreased in the RNAi lines (**Figure 6F**). Moreover, the *OsNPF7.3* OE lines accelerate soluble protein degradation under different nitrogen treatments during the later reproductive stages (Supplementary Figure 3). Nitrogen concentrates in the leaves and is transported to the panicles for seed growth. Therefore, leaf soluble protein content decreased with increasing *OsNPF7.3* expression and the translocation of nitrogen from leaf to panicle was accelerated. Consequently, more proteins were stored in the panicles, seed growth was enhanced, and grain yield increased.

### In Rice, *OsNPF7.3* OE Lines Enhance Grain Yield by Increase Tiller and Filled Grain Numbers in Rice

**FIGURE 7:**
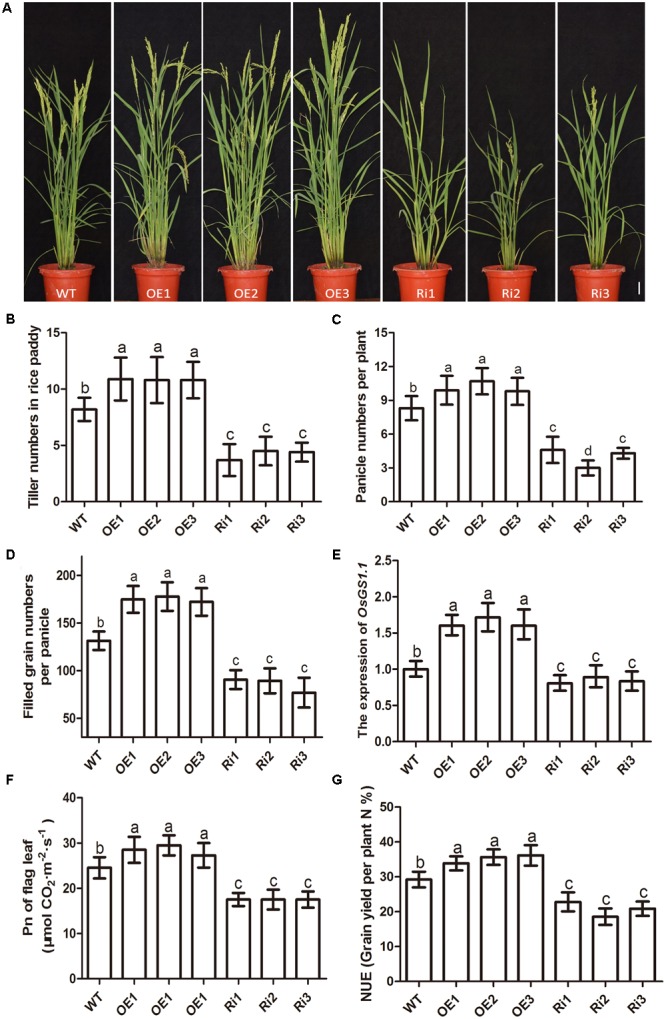
Agronomic trait analysis of paddy-grown *OsNPF7.3* transgenic plants. Phenotypes of paddy-grown wild-type (WT) and *OsNPF7.3*-overexpressing lines (OE1–OE3), and *OsNPF7.3*-RNAi lines (Ri1-Ri3) **(A)**. Tiller numbers **(B)**, panicles per plant **(C)**, filled grains per panicle **(D)**, the expression of OsGS1.1 **(E)**, photosynthesis analysis **(F)** and nitrogen utilization efficiency (NUE, **G**) were measured. The paddy rice grew at season June to October at rice experimental base of Huazhong Agricultural University under fertilizer (N/P/K = 19%/7%/14%) 180 kg/hm^2^. The plant number of each line is 30 for experiment. And the Error bars depict the SD from three independent experiments using three lines. Different letters in the columns indicate a significant difference of *P* < 0.05 according to Duncan’s test.

The effects of altered *OsNPF7.3* expression on agronomic traits associated with grain yield were evaluated using field-grown plants. The agronomic traits of three *OsNPF7.3* OE lines (OE1–OE3) were superior to those of the WT (**Figure 7A**). The three RNAi (Ri1–Ri3) lines presented with relatively lower tiller numbers, dwarfism, and short panicles (**Figure 7A**). The tiller numbers per plant (**Figure 7B**) and the panicle numbers per plant (**Figure 7C**) of the OE lines were greater than those of the WT and clearly greater in three of the lines. Nevertheless, the tiller and panicle numbers of the RNAi lines were distinctly lower (**Figures 7B,C**) than those of the WT. The filled grain numbers per panicle of the *OsNPF7.3* OE lines enhanced but apparently decreased in the RNAi line (**Figure 7D**). In addition, *OsNPF7.3* OE lines can clearly increase panicle length and secondary branch numbers per panicle but not affected primary branch number per panicle and 1000-grain weight (Supplementary Figure 4). The expression of N recycling related gene *OsGS1.1* increased in OE lines but decreased in RNAi lines (**Figure 7E**). Besides, the photosynthetic rate Pn (**Figure 7F**) and the nitrogen utilization efficiency (**Figure 7G**) of *OsNPF7.3* OE lines increased evidently in three lines relative to the WT but obviously decreased in RNAi lines compared to the WT. The grain yield traits were also measured under fertilizer regimes. Filled grain number per plant was significantly greater in the OE lines compared with the WT (**Table 1**). Furthermore, the higher grain yields of the three OE lines (over WT 5.5, 6.7, and 6.1%) were verified in a field test in 2016 (**Table 1**). Therefore, *OsNPF7.3* participates in rice grain yield maintenance by increasing tiller and filled grain numbers.

**Table 1:** Yield characteristics for WT and OE plants in paddy.

	WT	OE1	OE2	OE3
Effective panicles per plant	9.914 ± 1.729	10.925 ± 1.280^∗^	10.401 ± 2.060^∗^	10.617 ± 1.505^∗^
Biomass per plant (g)	241.26 ± 16.968	297.45 ± 25.560^∗^	290. 695 ± 20.028^∗^	287.45 ± 24.219^∗^
Filled grain number per plant	1524.629 ± 109.651	2034.508 ± 115.914^∗^	2080.125 ± 138.489^∗^	2051.845 ± 169.470^∗^
Grain weight per plant (g)	26.799 ± 4.382	31.213 ± 3.934^∗^	32.478 ± 3.585^∗^	31.034 ± 3.354^∗^
Actual yield increased over WT (%)		5.5	6.7	6.1

*For the field yield trials, the planting density was 19.98 cm × 19.98 cm and the number of rice plants was 100 for each OE line and WT ZH11. The data were analyzed using Duncan’s tests, ^∗^represents significant difference comparing to WT ZH11 (*P* < 0.05).*

## Discussion

Rice is one of the most important cereals worldwide (Sun et al., 2014), and contains 93 NPF family members (Léran et al., 2014). Rice low-affinity nitrate transporter OsNPF2.4, OsNPF6.5, and OsNPF2.2 are localized in the plasma membrane (Hu et al., 2015; Li et al., 2015; Xia et al., 2015). However, low-affinity nitrate transporter OsNPF7.2 is localized in the vacuolar membrane (Hu et al., 2016). Only two of the homologous peptide transporters genes in rice have been systematically studied. OsNPF4.1 (Li et al., 2009) and OsNPF8.20 (Fang et al., 2013) are localized in the plasma membrane but their substrates are still unknown. *OsNPF7.3* is unique and is the first peptide transporter gene identified in the rice NPF family to date. It was shown to transport di/tripeptides (Ouyang et al., 2010), but its localization in rice was unknown. We showed here that OsNPF7.3 is a vacuolar membrane localized transporter (**Figure 1**), and predicated with 12 TMs.

In rice, *OsNPF4.1* GUS signals intensified in the rachis, branches, stigmata, ovules, and the surfaces of the palea and the lemma during the young panicle elongation stage. In contrast, GUS activity was negligible in the roots, stems, and leaves of promoter-GUS transgenic plants (Li et al., 2009). *OsNPF8.20* expression was highest in the lateral roots, stems, and young panicles (Fang et al., 2013). Its expression pattern differs from that of *OsNPF4.1*. Here, we observed that the expression level of *OsNPF7.3* was higher in lateral roots stem, and young panicle (**Figure 2**). *OsNPF4.1* is specifically expressed in the phloem of vascular bundles (Li et al., 2009). Nevertheless, the expression of *OsNPF8.20* enriched in the cortical fiber cells of the lateral roots, and was clearly induced by ammonium (Fang et al., 2013). *Arabidopsis* NPF gene expression varies in response to N supply and N starvation (Sasaki et al., 2010; Xu et al., 2012). Unlike *OsNPF8.20*, the expression of *OsNPF7.3* is especially apparently regulated by organic nitrogen (**Figure 3**). The reason is that plants can modulate their development in response to nutrient availability (Puig et al., 2012). The staining gradually became lighter after 8 h with the ammonium and ammonium nitrate treatments in the root tips and lateral roots but kept to 24 h with the nitrate treatment. This observation may be explained by ammonium could be assimilated at the roots, and its assimilation is regulated by amino acids in the roots when the ammonium is full in plant (Li et al., 2015). Sectioning confirmed that higher expression of *OsNPF7.3* was observed in the root cerebral cortex and vascular bundle (**Figures 4A–C**), and becomes highly concentrated in the parenchyma cells and sieve cells in response to organic nitrogen application (**Figure 4**). It indicated that *OsNPF7.3* participates in nitrogen regulation. These results suggest that while the roles of *OsNPF4.1, OsNPF8.20*, and *OsNPF7.3* differ in rice, they are all classified as peptide transporters. The vascular system of a plant is a network of cells that interconnects all major plant organs (De Rybel et al., 2016). Vascular tissue formation in plants is a process with broad developmental and physiological consequences. Higher expression of *OsNPF7.3* was observed in the procambium, cerebral cortex and vascular bundle of root, and vascular bundle of stem (**Figure 4**). These observations indicate that *OsNPF7.3* translocates organic nitrogen in the root and loads organic nitrogen from the parenchyma cells to the vascular bundle. So *OsNPF7.3* facilitate to nitrogen translocation from the root to the leaf.

Over-expression of *OsNPF7.3* in rice may increase plant height depending on the nitrogen source and supply (Fan et al., 2014). Here, we indicated that altered expression of *OsNPF7.3* could affect dry weights and tiller numbers per plant in response to nitrogen treatment of OE and Ri lines (**Figure 5**). To determine why the RNAi of *OsNPF7.3* affects rice growth, we found that the free amino acid concentration in RNAi plants was higher in the leaf sheaths than in other organs (Supplementary Table 2). This observation indicates that knock down of *OsNPF7.3* may disrupt nitrogen balance and allocation in parenchyma cells in the stem and affect rice growth. However, free amino acid concentration was enhanced in root and leaf of *OsNPF7.3* OE lines, and this maybe more OsNPF7.3 can store nitrogen in the vacuole, then was used by plants for growth and development, especially the nitrogen mobilization from root to leaf through with leaf sheath. Likely, Knockout of vacuolar membrane localized transporter OsNPF7.2 could also retards rice growth (Hu et al., 2016). Leaves are N sinks during the vegetative stage. In the reproductive phase, the N is remobilized to the developing seeds (Zhang et al., 2010). In rice and wheat, up to 80% of the grain N is derived from the leaves (Mae and Ohira, 1981; Kichey et al., 2007; Tabuchi et al., 2007). The phloem of higher plants is an extensive conduit for the long-distance transport of many different compounds (van Bel et al., 2013; Jørgensen et al., 2015). During the reproductive phase in rice, the leaf is a major source and the panicle is an important sink (Masclaux-Daubresse et al., 2008). The expression of *OsNPF7.3* in the leaf and panicle phloem corroborates the hypothesis that *OsNPF7.3* participates in phloem discharge. We found that in the reproductive phase, the movement of the soluble protein in the leaves of *OsNPF7.3* OE lines is faster than that of the wild-type but slower than that of the RNAi lines. The soluble protein in the panicle and the total protein content per panicle were higher in *OsNPF7.3* OE lines than the WT (**Figure 6**). Therefore, *OsNPF7.3* influenced organic nitrogen transport from the leaves to the grains. Alterations in the expression of *OsNPF7.3* cause changes in N translocation, leaf N recycling, and grain numbers.

The agronomic traits tiller and filled grain numbers in all three paddy-grown *OsNPF7.3* OE lines were higher than those of the WT. Therefore, *OsNPF7.3* has potential application in increasing of nitrogen utilization efficiency and grain yield by altering its expression level.

## Author Contributions

ZF and MZ designed the research. ZF, GB, WH, and ZW performed the experiments. ZF, MZ, and XW drafted the manuscript.

## Conflict of Interest Statement

The authors declare that the research was conducted in the absence of any commercial or financial relationships that could be construed as a potential conflict of interest.

## Footnotes

1 http://www.cambia.org
