# Supplementary material for: The Rice Peptide Transporter OsNPF7.3 Is Induced by Organic Nitrogen, and Contributes to Nitrogen Allocation and Grain Yield
Source: Front Plant Sci. 2017 Aug 2;8:1338. doi: 10.3389/fpls.2017.01338 (PMC5539172; doi:10.3389/fpls.2017.01338)
Supplement: Supplementary file 1 [file Data_Sheet_1.DOCX]

Supplementary Material

The rice peptide transporter *OsNPF7.3* is induced by organic nitrogen, and contributes to nitrogen allocation and grain yield

*Zhongming Fang*^1, 2^*, Genxiang Bai*^1^*, Weiting Huang*^1^, *Zhixin Wang*^2^*, Mingyong Zhang*^3*^ and *Xuelu Wang*^2*^

^1^*Center of Applied Biotechnology*, *Wuhan Institute of Bioengineering*, *Wuhan 430415*, *China*

^2^*National Key Laboratory of Crop Genetic Improvement*, *Huazhong Agricultural University*, *Wuhan 430070*, *China*

^3^*Key Laboratory of South China Agricultural Plant Molecular Analysis and Genetic Improvement & Guangdong Provincial Key Laboratory of Applied Botany*, *South China Botanical Garden*, *Chinese Academy of Sciences*, *Guangzhou 510650*, *China*

***Corresponding author**Mingyong Zhang
zhangmy@scbg.ac.cn

Xuelu Wang
xlwang@mail.hzau.edu.cn

# Supplementary Data


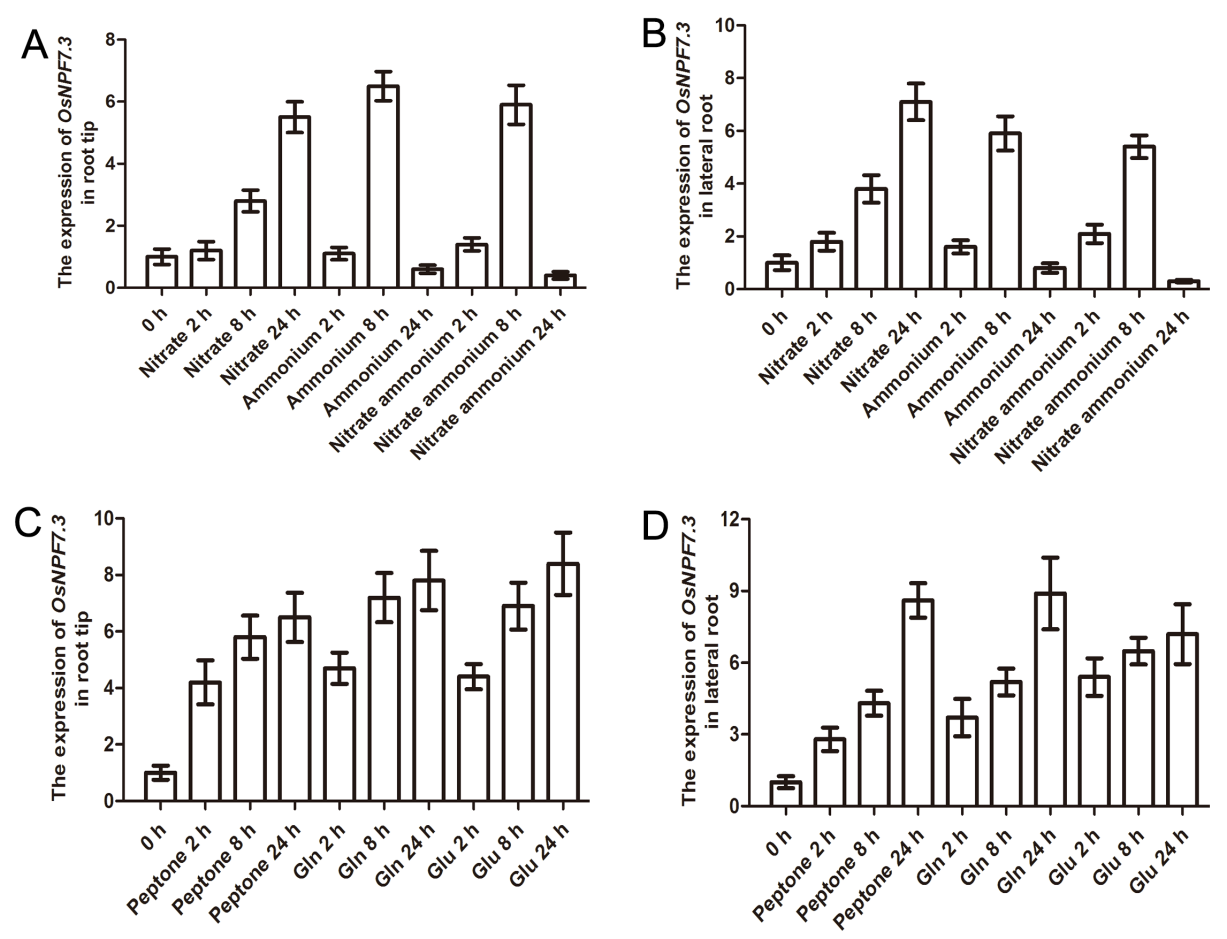


**Supplementary Figure 1 The expression of *OsNPF7.3* is regulated by** **both inorganic and organic nitrogen**

The expression of *OsNPF7.3* in root tip (A) and lateral root (B) in inorganic treatments, and the expression of *OsNPF7.3* in root tip (C) and lateral root (D) in organic treatments were measured. The ZH11 seedlings were grown for three weeks in basic nutrient solution with 1 mM NH_4_NO_3_ as the N source then transferred to N-free basic nutrient solution for three days (N starvation). The N-starved seedlings were transferred to basic nutrient solution supplemented with 2.0 mM NaNO_3_ or 1.0 mM (NH_4_)_2_SO_4_ or 1.0 mM NH_4_NO_3_ or supplemented with 2% peptone or 2.0 mM Gln or 2.0 mM Glu as the N sources.


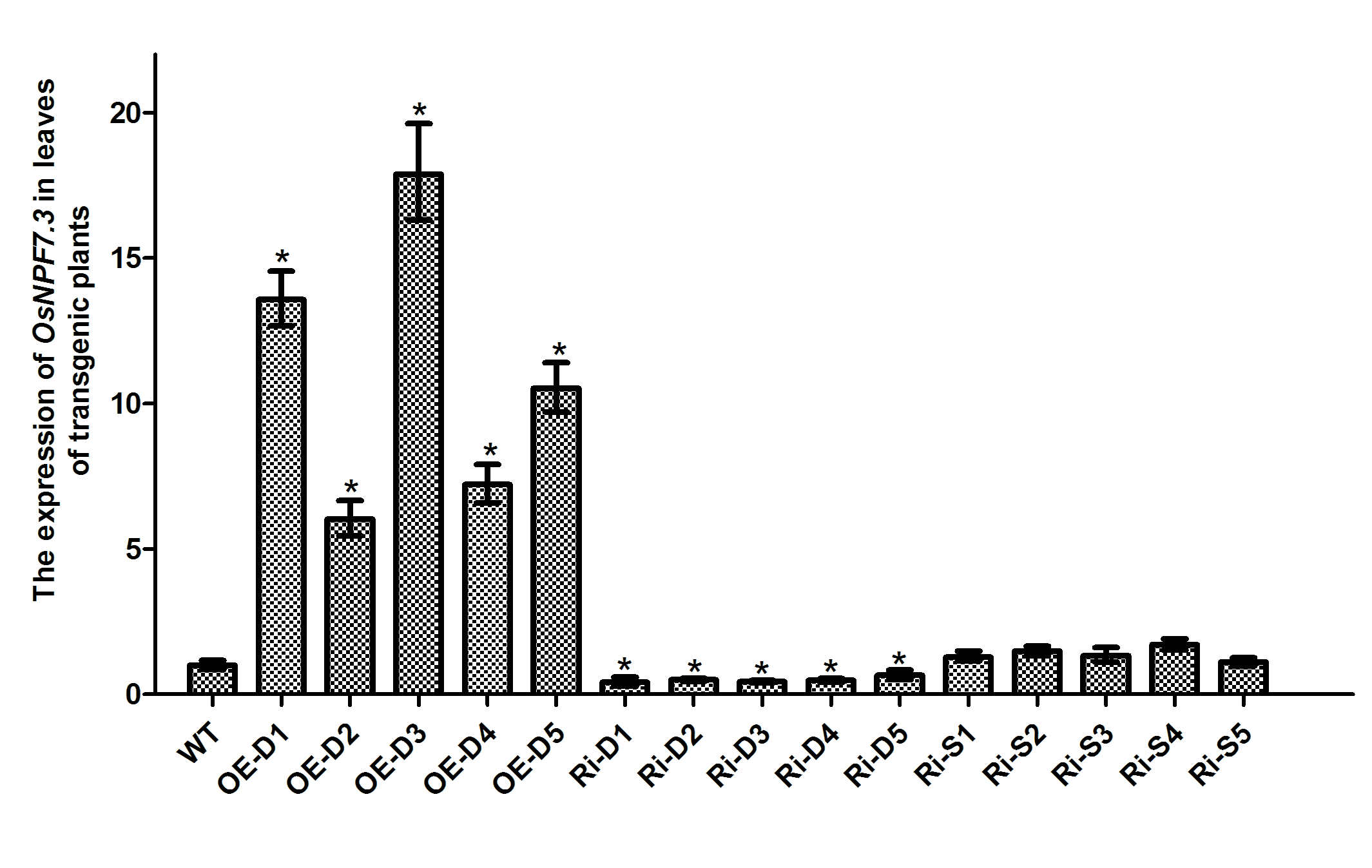


**Supplementary Figure 2 Molecular characterization of rice lines with altered *OsNPF7.3* expression**

Quantitative RT-PCR analysis of *OsNPF7.3* expression in the leaves of the wild type (WT), the *OsNPF7.3-*over-expressing (OE-D1 to OE-D5) lines, the *OsNPF7.3*-RNAi (Ri-D1 to Ri-D5) lines and the wild types isolated from *OsNPF7.3*-RNAi (Ri-S1to Ri-S5) lines that grown in paddy.

**
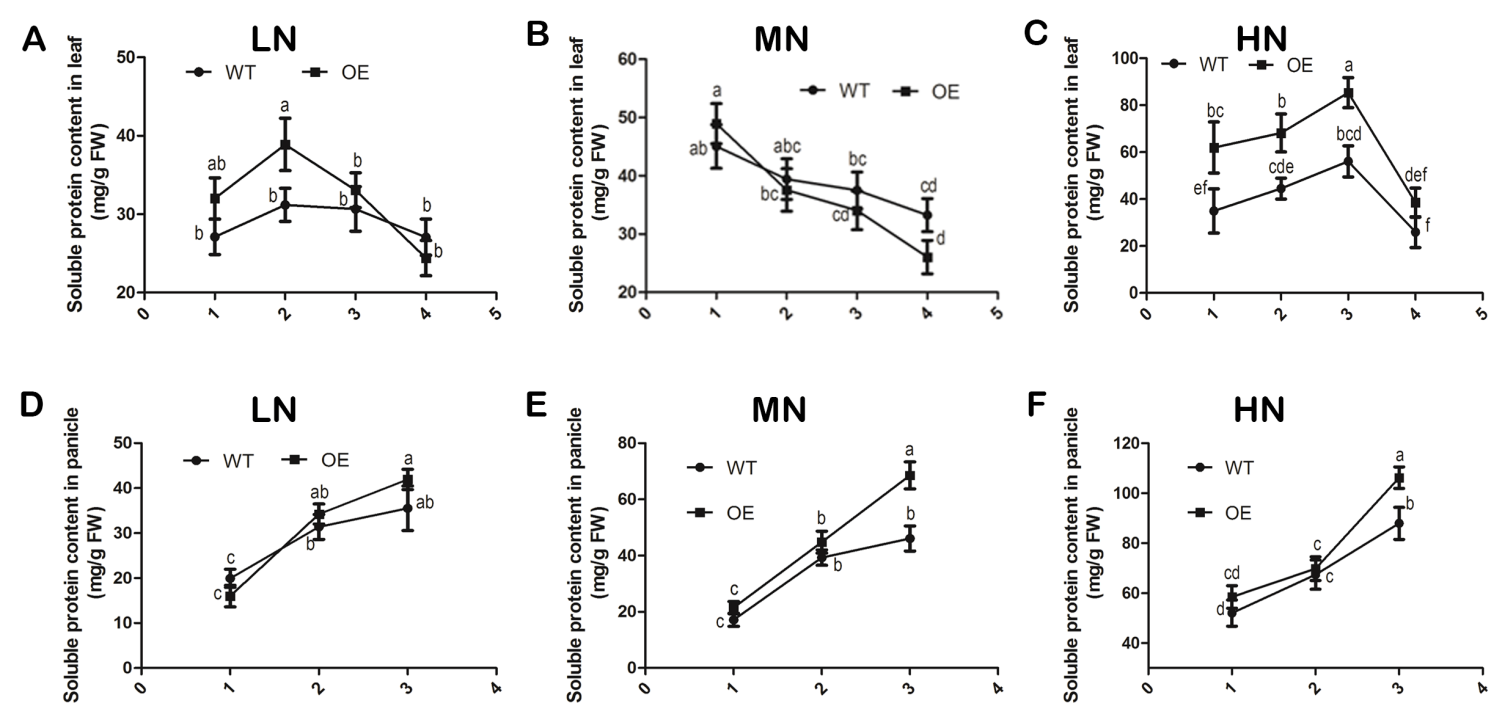
**

**Supplementary Figure 3 Over-expression of *OsNPF7.3* could accelerate soluble protein content from leaf to panicle**

Soluble protein contents were measured in leaves at 0.25 mM NH_4_NO_3_(LN, A), or 1 mM NH_4_NO_3_(MN, B), or 2.5 mM NH_4_NO_3_ (HN, C) at plant booting stage (1), heading stage (2), filling stage (3) and mature stage (4). Soluble protein contents were measured in panicles at 0.25 mM NH_4_NO_3_(LN, D), or 1 mM NH_4_NO_3_(MN, E), or 2.5 mM NH_4_NO_3_ (HN, F) at plant booting stage (1), heading stage (2), filling stage (3). Error bars depict the SD from three independent experiments. Different letters on the columns indicate a significant difference of P < 0.05 by Duncan test.


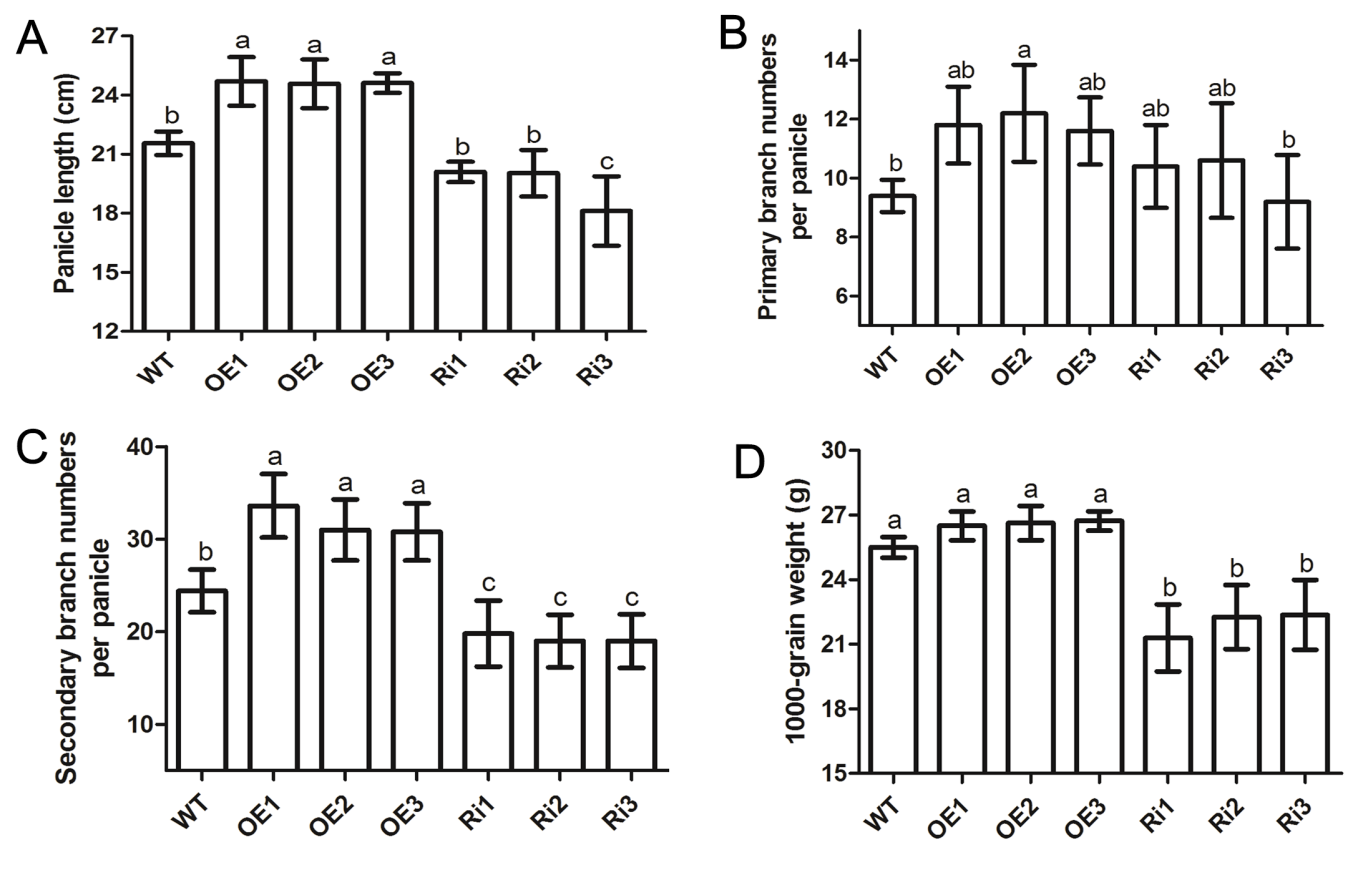


**Supplementary Figure 4 Agronomic trait analysis of paddy-grown *OsNPF7.3* transgenic plants**

Phenotypes of paddy-grown wild-type (WT) and *OsNPF7.3*-overexpressing lines (OE1-OE3), and *OsNPF7.3*-RNAi lines (Ri1-Ri3). Panicle length (A), primary branch numbers per panicle (B), secondary branch numbers per panicle (C), and 1000-grain weight (D) were measured. Error bars depict the SD from three independent experiments using three lines. Different letters in the columns indicate a significant difference of *P* < 0.05 according to Duncan’s test.

**Supplementary Table 1** **List of the realtime quantitative RT-PCR primers used in this study**

| **Name** | **Sequence (5' - 3')^a^** | **Note** |
| --- | --- | --- |
| ***OsNPF7.3* promoter** | | |
| Promoter-GUS-1F | GAATTCGAAGTGTATCTACAGCCAGGAA | For construction of plasmid *pOsNPF7.3*-*GUS* |
| Promoter-GUS-1R | CCATGGAGAGGCAAGCAAGCAAGG |  |
| Promoter-GUS-2F | CGTTTCGATGCGGTCACT | For detection of promoter(*OsNPF7.3*) -GUS transgenic plants. The length of fragment was 471 bp. |
| Promoter-GUS-2R | TTGCCAGAGGTGCGGATT |  |
| ***OsNPF7.3 -GFP* fusion** | | |
| FDF | AGATCTATGGACGCCGGCGAAATCATCGTG | For amplification of *OsNPF7.3* cDNA fragment to construct plasmid *pOsNPF7.3-GFP* |
| FDR | ACTAGTCGACACGACCAGCTTCACC |  |
| cDNA-*GFP*-F | AGAGGGTGAAGGTGATGC | For detection of *OsNPF7.3-*cDNA-*GFP* transgenic plants. The length of fragment was 421 bp. |
| cDNA-*GFP*-R | GATGTTGTGGCGGGTCTT |  |
| ***OsNPF7.3* overexpressing rice** | | |
| FEF | AGATCTATGGACGCCGGCGAAATCATCGTG | For amplification *OsNPF7.3* cDNA fragment to construct plasmid *p35S*-*OsNPF7.3* |
| FER | CTTAAGTCACGACACGACCAGCTTCACC |  |
| OE-F | GATGTTGGCGACCTCGTATT | For detection of *OsNPF7.3* over-expressing transgenic plants. The length of fragment was 517 bp. |
| OE-R | TCGTTATGTTTATCGGCACTTT |  |
| ***OsNPF7.3* RNAi rice** | | |
| FIF | ACTAGTAACCTTGCTTGCTTGCCTCT | Amplification the reverse fragment of *OsNPF7.3* cDNA to construct plasmid *p OsNPF7.3i* |
| FIR | GAGCTCGCTTTGCTCCTCGCTTTCT |  |
| FIF | GGTACCAACCTTGCTTGCTTGCCTCT | Amplification the forward fragment of *OsNPF7.3* cDNA to construct plasmid *p OsNPF7.3i* |
| FIR | GGATCCGCTTTGCTCCTCGCTTTCT |  |
| RNAi-F | ATGTTGGCGACCTCGTATT | For detection of *OsNPF7.3* RNAi transgenic plants. The length of fragment was 515 bp. |
| RNAi-R | CGTTATGTTTATCGGCACTTT |  |
| **qRT-PCR** | | |
| Actin1-q-F | CGGTGTCATGGTCGGAAT | For q-PCR of *OsActin1* (LOC_Os03g50885). |
| Actin1-q-R | GCTCGTTGTAGAAGGTGT |  |
| OsNPF7.3-q-F | ACATCGCGCAGCTGGAGTTC | For q-quantitative and q-PCR of *OsNPF7.3* |
| OsNPF7.3-q-R | AGTCCATGTGTCCTTCGTTGAGGT |  |
| OsGS1.1-q-F | CACCAACAAGAGGCACAATG | For q-quantitative and q-PCR of *OsNPF7.3* |
| Os GS1.1-q-R | ACTCCCACTGTCCTGGCAT |  |

**Supplementary Table 2** **Amino acid concentration (mg g^-1^ DW) of rice seedlings of different transgenic plant grown in paddy**

|  | WT | OE1 | OE2 | Ri1 | Ri2 |
| --- | --- | --- | --- | --- | --- |
| Root |  |  |  |  |  |
| Asp | 0.591±0.182 | 0.827±0.070* | 0.667±0.233 | 0.478±0.094 | 0.674±0.035 |
| Thr | 0.728±0.211 | 1.353±0.045* | 0.984±0.274 | 0.758±0.125 | 0.809±0.120 |
| Ser | 0.631±0.172 | 1.194±0.080* | 1.089±0.625* | 0.723±0.145 | 0.713±0.065 |
| Glu | 0.570±0.026 | 0.587±0.071 | 0.619±0.008 | 0.487±0.120 | 0.659±0.029 |
| Gly | 0.867±0.070 | 1.224±0.124* | 1.106±0.363* | 0.880±0.050 | 0.808±0.058 |
| Ala | 1.658±0.108 | 2.241±0.150* | 2.152±0.396* | 1.389±0.271* | 1.283±0.024* |
| Cys | 0.146±0.020 | 0.109±0.145 | 0.162±0.016 | 0.161±0.044 | 0.124±0.032 |
| Val | 0.627±0.118 | 0.766±0.164 | 0.807±0.254 | 0.631±0.159 | 0.642±0.023 |
| Met | 0.055±0.035 | 0.069±0.017 | 0.048±0.012 | 0.103±0.094* | 0.036±0.019 |
| Ile | 0.116±0.001 | 0.155±0.018* | 0.135±0.026 | 0.108±0.010 | 0.143±0.010* |
| Leu | 0.321±0.007 | 0.372±0.032 | 0.345±0.057 | 0.285±0.010 | 0.256±0.018 |
| Tyr | 0.277±0.009 | 0.256±0.020 | 0.230±0.003 | 0.228±0.022 | 0.248±0.015 |
| Phe | 0.154±0.015 | 0.196±0.032 | 0.179±0.030 | 0.154±0.003 | 0.183±0.014 |
| Lys | 0.340±0.006 | 0.440±0.038 | 0.396±0.087 | 0.292±0.053 | 0.243±0.026 |
| His | 0.140±0.024 | 0.183±0.019* | 0.164±0.041* | 0.122±0.012 | 0.145±0.005 |
| Arg | 0.275±0.023 | 0.370±0.043* | 0.341±0.111* | 0.238±0.043* | 0.233±0.024* |
| Pro | 0.180±0.020 | 0.210±0.023 | 0.194±0.006 | 0.182±0.023 | 0.196±0.030 |
| Total | 7.675±0.251 | 10.552±0.934* | 9.617±3.101* | 7.216±0.293 | 7.397±0.130 |
| Leaf Sheath |  |  |  |  |  |
| Asp | 2.328±0.283 | 2.759±0.247 | 2.917±0.234 | 5.788±0.295* | 4.282±0.181* |
| Thr | 6.877±0.266 | 8.783±0.060* | 11.187±0.440* | 8.652±0.440* | 8.859±0.407* |
| Ser | 8.498±0.109 | 9.451±0.196* | 10.817±0.650* | 10.244±0.223* | 10.139±0.515* |
| Glu | 1.930±0.028 | 2.102±0.132 | 1.932±0.080 | 2.821±0.200* | 2.018±0.014 |
| Gly | 2.501±0.052 | 2.589±0.083 | 3.702±0.180* | 3.695±0.222* | 3.209±0.066* |
| Ala | 20.264±0.146 | 22.054±1.194 | 26.113±1.401* | 27.149±0.518* | 24.832±0.306* |
| Cys | 0.561±0.044 | 0.560±0.043 | 0.511±0.089 | 0.775±0.058* | 0.621±0.029* |
| Val | 4.145±0.072 | 4.675±0.325 | 4.301±0.226 | 6.325±0.219* | 6.319±0.146* |
| Met | 1.668±0.031 | 1.125±0.388 | 1.029±0.047 | 1.022±0.007 | 1.832±0.062 |
| Ile | 0.907±0.020 | 1.044±0.091 | 0.906±0.047 | 1.358±0.038* | 1.159±0.035 |
| Leu | 2.503±0.058 | 2.709±0.126 | 2.389±0.118 | 3.222±0.095* | 3.036±0.060* |
| Tyr | 2.358±0.063 | 2.130±0.191 | 2.057±0.094 | 2.894±0.043* | 3.137±0.059* |
| Phe | 2.110±0.018 | 2.037±0.073 | 1.992±0.107 | 3.115±0.111* | 2.984±0.040* |
| Lys | 0.789±0.025 | 0.960±0.139 | 1.048±0.047 | 1.307±0.030* | 0.904±0.019 |
| His | 0.564±0.041 | 0.628±0.030 | 0.627±0.022 | 1.008±0.032* | 1.026±0.023* |
| Arg | 1.836±0.029 | 2.010±0.105 | 1.974±0.106 | 2.781±0.146* | 2.842±0.107* |
| Pro | 3.363±0.076 | 2.916±0.319 | 2.656±0.127 | 3.359±0.040 | 3.561±0.103 |
| Total | 63.204±1.094 | 68.532±1.454* | 76.159±3.701* | 85.513±1.822* | 80.759±1.900* |
| Leaf |  |  |  |  |  |
| Asp | 3.766±0.389 | 7.713±0.145* | 7.326±0.330* | 3.661±0.113 | 3.703±0.165 |
| Thr | 8.947±0.542 | 10.711±0.512* | 11.331±0.332* | 8.259±0.789 | 8.733±0.114 |
| Ser | 9.389±0.336 | 8.071±0.087 | 14.041±0.241* | 9.781±0.629 | 9.831±0.446 |
| Glu | 7.773±0.629 | 10.418±0.265* | 11.467±0.143* | 6.677±0.239 | 7.264±0.348 |
| Gly | 4.045±0.364 | 5.756±0.147* | 4.075±0.026 | 3.744±0.063 | 3.872±0.073 |
| Ala | 30.671±0.872 | 38.885±0.851* | 31.630±1.299 | 30.446±0.286 | 30.562±0.267 |
| Cys | 1.108±0.152 | 1.060±0.088 | 1.026±0.041 | 1.006±0.042 | 0.978±0.066 |
| Val | 7.287±0.207 | 8.925±0.195* | 9.312±0.212* | 6.463±0.212* | 6.526±0.194* |
| Met | 2.639±0.427 | 2.932±0.079 | 3.377±0.251 | 3.047±0.068 | 3.191±0.121 |
| Ile | 1.585±0.161 | 1.962±0.046* | 1.976±0.090* | 1.728±0.277 | 1.60±0.261 |
| Leu | 3.062±0.272 | 3.581±0.084* | 3.045±0.142 | 2.966±0.413 | 2.476±0.101* |
| Tyr | 2.426±0.473 | 3.148±0.078* | 2.800±0.120 | 2.732±0.276 | 2.704±0.221 |
| Phe | 3.053±0.175 | 3.178±0.076 | 3.449±0.174* | 3.224±0.435 | 2.695±0.101* |
| Lys | 1.788±0.175 | 2.672±0.058* | 1.908±0.102 | 1.674±0.109 | 1.824±0.119 |
| His | 1.488±0.168 | 1.558±0.036 | 1.759±0.108 | 1.319±0.019 | 1.358±0.040 |
| Arg | 2.378±0.249 | 3.036±0.063* | 2.302±0.172 | 2.089±0.284 | 2.430±0.332 |
| Pro | 2.655±0.299 | 2.989±0.157 | 2.488±0.275 | 1.899±0.165* | 1.274±0.216* |
| Total | 94.059±3.821 | 116.592±2.032* | 113.313±1.912* | 90.717±1.674 | 91.027±0.965 |
